# *Curcuma longa* L. extract exhibits anti-inflammatory and cytoprotective functions in the articular cartilage of monoiodoacetate-injected rats

**DOI:** 10.29219/fnr.v68.10402

**Published:** 2024-03-19

**Authors:** Hyelim Kim, Jaeeun Jung, Minhee Lee, Minha Kim, Namgil Kang, Ok-Kyung Kim, Jeongmin Lee

**Affiliations:** 1 Department of Medical Nutrition, Graduate School of East-West Medical Science, Kyung Hee University, Yongin 17104, Republic of Korea; 2 Department of Food Innovation and Health, Graduate School of East-West Medical Science, Kyung Hee University, Yongin 17104, Republic of Korea; 3 Nutrione Co., Ltd, Seoul 05510, Republic of Korea; 4 Division of Food and Nutrition and Human Ecology Research Institute, Chonnam National University, Gwangju, 61186, Republic of Korea

**Keywords:** *osteoarthritis*, Curcuma longa L, *MMPs*, *chondrocytes*

## Abstract

**Background:**

Osteoarthritis (OA), the most prevalent form of arthritis, is a degenerative joint disease marked by the progressive deterioration of articular cartilage, leading to clinical manifestations such as joint pain.

**Objective:**

This study investigated the effects of *Curcuma longa* L. extract (CL) containing curcumin, demethoxycurcumin, and bisdemethoxycurcumin on monosodium iodoacetate (MIA)-induced OA rats.

**Design:**

Sprague–Dawley rats with MIA-induced OA received CL supplementation at doses of 5, 25, and 40 mg/kg body weight.

**Results:**

CL extract administration suppressed mineralisation parameters and morphological modifications and decreased arachidonate5-lipoxygenase and leukotriene B4 levels in articular cartilage. Additionally, it decreased serum prostaglandin E2, NO, and glycosaminoglycanlevels as well as the protein expression of phosphorylated inhibitor kappa B-alpha, phosphorylated p65, cyclooxygenase-2, and inducible nitric oxide synthase in the cartilage of MIA-injected rats. Furthermore, it also reduced matrix metalloproteinases and elevated SMAD family member 3 phosphorylation, tissue inhibitor of metalloproteinases, aggrecan, collagen type I, and collagen type II levels in the articular cartilage of MIA-induced OA rats.

**Conclusions:**

This study’s findings suggest that CL supplementation helps prevent OA development and is an effective therapy for OA.

## Popular scientific summary

- Curcuma longa L. extract (CL) effectively ameliorated histological and mineralisation-parameter alterations, pain intensity, and inflammation and suppresses the degradation of articular cartilage in osteoarthritis (OA) rats.
- Our findings suggest that CL supplementation may prevent OA development and exhibit effectiveness in OA therapies.

Osteoarthritis (OA) is a degenerative joint disorder characterised by the progressive breakdown of cartilage in the joints, leading to pain, stiffness, and impaired mobility. It is the most prevalent form of arthritis and a major cause of disability, particularly among older adults (1, 2). The primary cause of OA is often attributed to the wear and tear of joints over time, although various contributing factors exist, including genetic predisposition, joint injuries, and obesity. In OA, the protective cartilage that cushions the ends of bones within joints gradually deteriorates, causing bones to rub against each other, resulting in pain and reduced joint function. Common OA symptoms include joint pain, swelling, and a diminished range of motion. While OA can affect any joint, it predominantly impacts weight-bearing joints, such as the knees, hips, and spine (1–3).

OA development involves intricate pathways related to the synthesis and degradation of articular cartilage. In a healthy joint, chondrocytes, the specialised cells residing in cartilage, maintain a delicate balance between the synthesis and degradation of extracellular matrix (ECM) components, primarily collagen and proteoglycans (4–6). However, in OA, this equilibrium is disrupted. The increased mechanical stress on the joint, along with factors such as aging and genetic predisposition, triggers a cascade of events. In response to these stimuli, the chondrocytes undergo phenotypic modifications, producing an altered matrix that is more susceptible to degradation. Matrix metalloproteinases (MMPs) and aggrecanases, enzymes upregulated in OA, contribute to collagen and proteoglycan breakdown, leading to the loss of cartilage integrity. Consequently, the cartilage undergoes progressive deterioration, exposing the underlying bone and causing pain, inflammation, and functional impairment characteristic of OA (7, 8).

Previous studies, including animal and human trials, have demonstrated promising results suggesting that *Curcuma longa* L. (CL), commonly known as turmeric, possesses anti-inflammatory and anti-arthritic properties for arthritis prevention and treatment (9, 10). The active compound in CL, curcumin, has been the focus of various studies exploring its potential therapeutic effects on arthritis. Curcumin is recognised for its ability to modulate inflammatory pathways, inhibit the activity of inflammatory enzymes, and reduce oxidative stress (11, 12). Here, we explored the effect of CL. extract containing curcumin, demethoxycurcumin, and bisdemethoxycurcumin on alterations in bone mass, bone microstructure, and exercise performance in rats with monosodium iodoacetate (MIA)-induced OA to validate its efficacy. Moreover, we determined the factors involved in the synthesis and degradation of articular cartilage and inflammation.

## Materials and methods

### Extract and animals

**Fig. 1:**
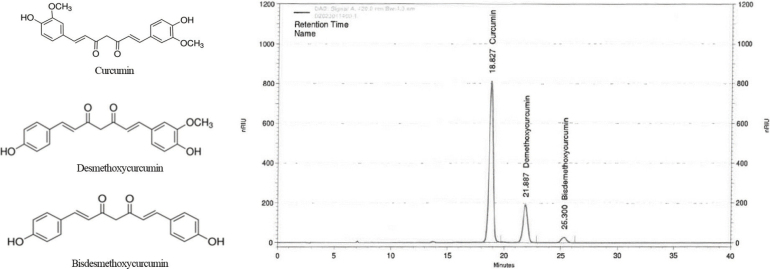
High-performance liquid chromatography analysis of the curcumin, demethoxycurcumin, and bisdemethoxycurcumin levels in CL. CL = *Curcuma longa* L. extract.

Water-dispersible *Curcuma longa* L. extract (CL; TurmXTRA 60N^®^) containing 60% curcuminoids (Fig. 1) was provided by Nutrione (Seoul, Korea). Sprague–Dawley rats (4-week-old males) were obtained from SaeRon Bio (Uiwang, Korea) and housed in cages with an automatically controlled environment (temperature: 22 ± 2°C; humidity: approximately 50%; lighting: 12-h light–dark cycle). The rats were randomly categorised into six groups of eight mice each as follows: normal control (AIN93G diet), control (C; AIN93G diet and MIA injection), positive control (PC; AIN93G diet containing ibuprofen at 20 mg/kg body weight [bw] and MIA injection), and three experimental groups receiving MIA injection and an AIN93G diet containing CL at different concentrations: CL5 (5 mg/kg/bw), CL25 (25 mg/kg/bw), and CL40 (40 mg/kg/bw). Three days after dietary supplementation, the rats were anesthetised with isoflurane and administered MIA (50 μL, 60 mg/mL) (Sigma–Aldrich) via a single injection into the right knee joint. The normal group was administered 0.9% saline via injection. The rats were sacrificed via cervical dislocation 31 days after MIA injection and dietary supplementation. The experimental protocol for this animal study was approved by the Institutional Animal Care and Use Committee of Kyung Hee University (KHGASP-23-098).

### Hematoxylin and eosin staining

Knee joints from the rats were fixed with 10% neutral buffered formaldehyde solution to preserve their structure. The fixed sample was subsequently dehydrated with graded ethanol (decreasing concentrations of 100–70%) and embedded in paraffin wax. The paraffin blocks were sliced into 7-μm sections, stained with Hematoxylin and eosin (H&E), washed with distilled H_2_O, and observed using an optical microscope to visualise knee joints.

### Micro-computed tomography

Micro-computed tomography (CT) imaging of the formalin-fixed articular cartilage from the rats was used to measure the roughness of the bone surface. Micro-CT image scanning was conducted using the Skyscan 1172® X-ray μCT Scanning System (Bruker, Belgium). After standardised reconstruction of the scanned images, each sample’s data were generated using micro-CT software to orient each sample in the same manner.

### Treadmill test

Four weeks post-MIA injection, we used a rat-specific treadmill (Jeollanamdo Institute of Natural Resources Research, Korea) to measure rear pressure, rear propulsion, and running speed.

### Enzyme-linked immunosorbent assay analysis

The levels of arachidonate 5-lipoxygenase (5-LOX) were measured using the Lipoxygenase Inhibitor Screening Assay kit (Cayman chemical, Ann Arbor, MI, USA). The levels of glycosaminoglycan (GAG) were measured using an enzyme-linked immunosorbent assay (ELISA) kit (Mybiosource, San Diego, CA, USA). The levels of leukotriene B4 (LTB_4_), prostaglandin E2 (PGE_2_), and nitric oxide (NO) were measured using an ELISA kit (R&D Systems, Minneapolis, MN, USA). The assays were conducted according to the manufacturer’s instructions.

### Western blot analysis

Total protein was extracted from chondrocytes and cartilage tissue using 4X NuPAGE™ LDS Sample Buffer (Life Technologies, Gaithersburg, MD, USA). Protein samples containing 20–100 μg of protein from cells were separated using gel electrophoresis and transferred to membranes. The membranes were blocked and subsequently incubated with primary antibodies (SMAD family member 3 [Smad-3; Cell Signaling Technology (CST), Danvers, MA, USA], phosphorylated Smad-3 [CST], MMP-2 [Abcam, Waltham, MA, USA], MMP-3 [Abcam], MMP-9 [Abclonal Science Inc., Woburn, MA, USA], MMP-13 [Abcam], inhibitor kappa B-alpha [IκBα; CST], phosphorylated IκBα [CST], p65 [Abcam], phosphorylated p65 [CST], cyclooxygenase-2 [COX-2; CST], inducible nitric oxide synthase [iNOS; Abcam], and β-actin [Bethyl Laboratories Inc., Montgomery, TX, USA]) and secondary antibodies (Bethyl Laboratories Inc). Bands were captured using the ChemiDoc™ Imaging System (Bio-Rad, Hercules, CA, USA) and quantified using ImageJ software (version 1.53e; National Institutes of Health, Bethesda, MD, USA).

### Total RNA extraction and real-time polymerase chain reaction

Total RNA was extracted from chondrocytes and cartilage tissue using a commercial RNA extraction kit (QIAGEN, Gaithersburg, MD, USA). The extracted RNA was assessed for ratio and concentration using a NanoDrop™ spectrophotometer. Thereafter, complementary DNA (cDNA) was synthesised from purified total RNA (500 ng/mL) using the iScript^TM^ cDNA Synthesis Kit (Bio-Rad). Real-time polymerase chain reaction (PCR) was performed using a CFX Connect^TM^ Real Time System (Bio-Rad) with the iScript^TM^ Green Supermix, cDNA, and custom-designed primers (Table 1), and the real-time PCR reactions were run in duplicate. Data analysis was conducted using CFX Manager^TM^ 3.1 analysis software (Bio-Rad).

**Table 1:** Primer sets used for real-time RT-PCR

Gene	Forward sequence (5’-3’)	Reverse sequence (5’-3’)
*MMP-2*	TTG CTG GTG GCC ACA TTC T	GCC TCG TAC ACG GCA TCA A
*MMP-3*	GAG TGT GGA TTC TGC CAT TGA G	TTA CAG CCT CTC CTT CAG AGA
*MMP-9*	TCG AAG GCG ACC TCA AGT G	TTC GGT GTA GCT TTG GAT CCA
*MMP-13*	TGA TGG GCC TTC TGG TCT TCT	CCC CGC CAA GGT TTG G
*TIMP-1*	AAG GGC TAC CAG AGC GAT CA	ATC GAG ACC CCA AGG TAT TGC
*TIMP-3*	GAC CGA CAT GCT TC CAA TTT C	GCT GCA GTA GCC ACC CTT CT
*Aggrecan*	GAA GTG GCG TCC AAA CCA A	CGT TCC ATT CAC CCC TCT CA
*Collagen Type I*	GAG CGG AGA GTA CTG GAT CGA	CTG ACC TGT CTC CAT GTT GCA
*Collagen Type II*	GCA ACA GCA GGT TCA CGT ACA	TCG GTA CTC GAT GAT GGT CTT G
*COX2*	AGA GAA AGA AAT GGC TGC AGA GTT	AGC AGG GCG GGA TAC AGT
*iNOS*	TGG TGA AAG CGG TGT TCT TTG	ACG CGG GAA GCC ATG A
*TNF-*α	GGC TGC CCC GAC TAT GTG	CTC CTG GTA TGA AGT GGC AAA TC
*IL-1*β	CTG ACA GAC CCC AAA AGA TTA AGG	CTC ATC TGG ACA GCC CAA GTC
*IL-6*	GCC CTT CAG GAA CAG CTA TGA	TGT CAA CAA CAT CAG TCC CAA GA
*GAPDH*	ACC ACA GTC CAT GCC ATC AC	TCC ACC ACC CTG TTG CTG TA

RT-PCR = Real-time polymerase chain reaction; MMP = Matrix metalloproteinases; TIMP = tissue inhibitor of metalloproteinases; COX2 = cyclooxygenase-2; iNOS = inducible nitric oxide synthase; TNF-*α* = tumour necrosis factor-*α*.

### Statistical analysis

All data are expressed as the mean ± standard deviation (SD). Significant differences were determined using one-way analysis of variance (ANOVA) and Duncan’s multiple range test (SPSS PASW Statistics version 23.0; SPSS Inc., Chicago, IL, USA). Statistical significance was set at *P* < 0.05.

## Results

### CL ameliorated histological and mineralisation-parameter alterations in rats with MIA-induced OA

**Fig. 2:**
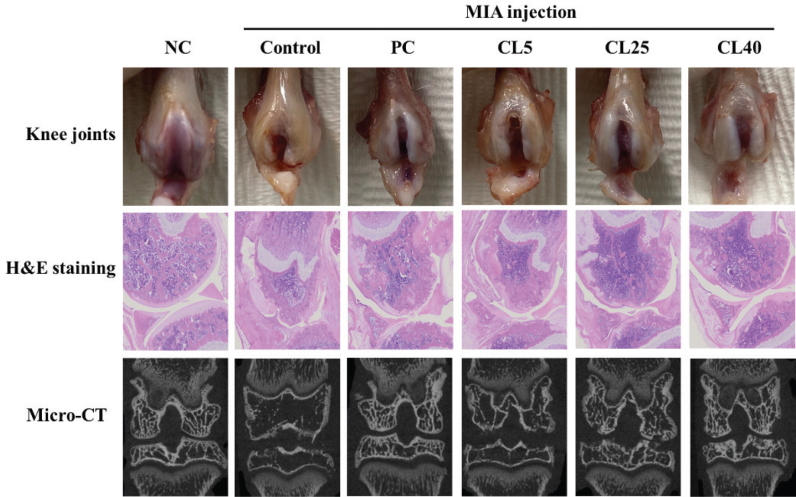
Effects of CL on the morphological and histological alterations in rats with MIA-induced osteoarthritis. NC: normal diet; Control (C): normal diet + MIA injection; PC: diet containing 20 mg/kg/bw ibuprofen + MIA injection; CL5: diet containing 5 mg/kg/bw CL + MIA injection; CL25: diet containing 25 mg/kg/bw CL + MIA injection; CL40: diet containing 40 mg/kg/bw CL + MIA injection. CL = *Curcuma longa* L. extract; MIA = monosodium iodoacetate; NC = Normal Control; PC = Positive control.

Pre-treatment with CL suppressed the death of chondrocytes in H_2_O_2_-treated cells (Supplementary Fig. 1). Therefore, we speculated that CL would be able to protect against cartilage damage in animal models. We observed morphological modifications characterised by the presence of cartilage fibrillation, fissuring, erosion, and matrix degradation in the knee joints of MIA-induced OA rats (Fig. 2). Additionally, MIA injection induced a decrease in bone mineral density, bone volume/total tissue volume, trabecular number, and trabecular thickness along with an increase in trabecular separation when knee joints were assessed using micro-CT (Table 2). In contrast, rats treated with ibuprofen or CL exhibited suppressed morphological alterations and mineralisation parameters, including bone mineral density, bone volume/total tissue volume, trabecular number, and trabecular thickness, and increased trabecular separation (*P* < 0.05; Fig. 2 and Table 1). These results indicate that dietary CL administration has a protective effect against MIA-induced cartilage tissue damage, potentially mitigating the degenerative effects of OA.

**Table 2:** Effects of CL on bone marrow density, bone volume/trabecular number, trabecular thickness, and trabecular separation in rats with MIA-induced osteoarthritis according to micro-CT

Measurements	MIA injection					
	NC	C	PC	CL5	CL25	CL40
Bone marrow density (mg/cc)	348.02 ± 17.36^a^	286.62 ± 4.85^b^	328.30 ± 11.82^a^	296.34 ± 7.36^b^	304.13 ± 10.69^b^	332.50 ± 16.50^a^
Bone volume/ Total tissue volume	0.48 ± 0.01^a^	0.33 ± 0.02^c^	0.45 ± 0.05^a^	0.34 ± 0.02^c^	0.34 ± 0.03^c^	0.38 ± 0.01^b^
Trabecular number	2.18 ± 0.07^a^	1.70 ± 0.02^e^	2.00 ± 0.09^b^	1.74 ± 0.01^de^	1.79 ± 0.02^cd^	1.85 ± 0.01^d^
Trabecular thickness (mm)	0.21 ± 0.01^a^	0.19 ± 0.01^b^	0.22 ± 0.004^a^	0.20 ± 0.01^ab^	0.20 ± 0.02^ab^	0.21 ± 0.01^a^
Trabecular separation (mm)	0.32 ± 0.04^d^	0.43 ± 0.01^a^	0.32 ± 0.02^d^	0.42 ± 0.02^ab^	0.38 ± 0.01^bc^	0.35 ± 0.02^cd^

NC: normal diet; Control (C): normal diet + MIA injection; PC: diet containing 20 mg/kg/bw ibuprofen + MIA injection; CL5: diet containing 5 mg/kg/bw CL + MIA injection; CL25: diet containing 25 mg/kg/bw CL + MIA injection; CL40: diet containing 40 mg/kg/bw CL + MIA injection. Values are presented as the mean + SD. Different letters (a > b > c > d) indicate significant differences with *P* < 0.05, as determined using Duncan’s multiple range test. CL = *Curcuma longa* L. extract; MIA = monosodium iodoacetate; CT = computed tomography; NC = Normal Control; PC = Positive Control.

### CL ameliorated pain intensity in rats with MIA-induced OA

**Fig. 3:**
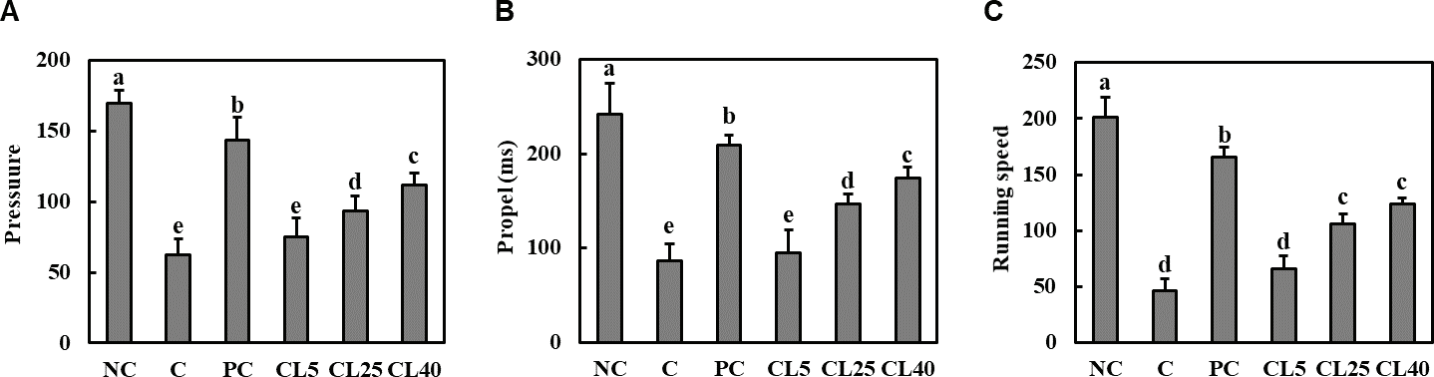
Effects of CL on the rear pressure (A), rear propulsion (B), and running speed (C) of rats with MIA-induced osteoarthritis. NC: normal diet; Control (C): normal diet + MIA injection; PC: diet containing 20 mg/kg/bw ibuprofen + MIA injection; CL5: diet containing 5 mg/kg/bw CL + MIA injection; CL25: diet containing 25 mg/kg/bw CL + MIA injection; CL40: diet containing 40 mg/kg/bw CL + MIA injection. Values are presented as the mean + SD. Different letters (a > b > c > d > e) indicate significant differences with *P* < 0.05, as determined using Duncan’s multiple range test. CL = *Curcuma longa* L. extract; MIA = monosodium iodoacetate; NC = Normal Control; PC = Positive control.

We measured the rear pressure, rear propulsion, and running speed of rats using a treadmill to confirm the effect of CL on pain intensity. We found that rear pressure, rear propulsion, and running speed significantly decreased in the MIA-induced OA group compared with those in the normal control group. However, dietary administration of ibuprofen, CL25, or CL40 to MIA-induced OA rats increased rear pressure, rear propulsion, and running speed compared with that of the control (*P* < 0.05; Fig. 3). Therefore, these findings suggest that dietary administration of CL potentially alleviates OA-associated pain.

### CL ameliorated serum levels of PGE_2_, NO, and GAG in rats with MIA-induced OA

**Fig. 4:**
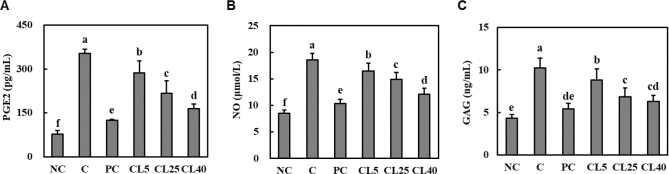
Effects of CL on serum PGE_2_ (A), NO (B), and GAG (C) in rats with MIA-induced osteoarthritis. Normal Control (NC): normal diet; Control (C): normal diet + MIA injection; Positive control (PC): diet containing 20 mg/kg/bw ibuprofen + MIA injection; CL5: diet containing 5 mg/kg/bw CL + MIA injection; CL25: diet containing 25 mg/kg/bw CL + MIA injection; CL40: diet containing 40 mg/kg bw CL + MIA injection. Values are presented as the mean + SD. Different letters (a > b > c > d > e > f) indicate significant differences with *P* < 0.05, as determined using Duncan’s multiple range test. CL = *Curcuma longa* L. extract; MIA = monosodium iodoacetate.

Figure 4 illustrates that MIA-injected rats exhibited significant increases in the serum levels of PGE_2_ and NO, compared with normal rats. Additionally, MIA-induced OA rats exhibited increased serum GAG levels in articular cartilage, indicating articular cartilage damage. However, the PGE_2_, NO, and GAG levels were significantly reduced in ibuprofen- and CL-treated rats compared with those in control rats (*P* < 0.05).

### CL ameliorated inflammation in rats with MIA-induced OA

**Fig. 5:**
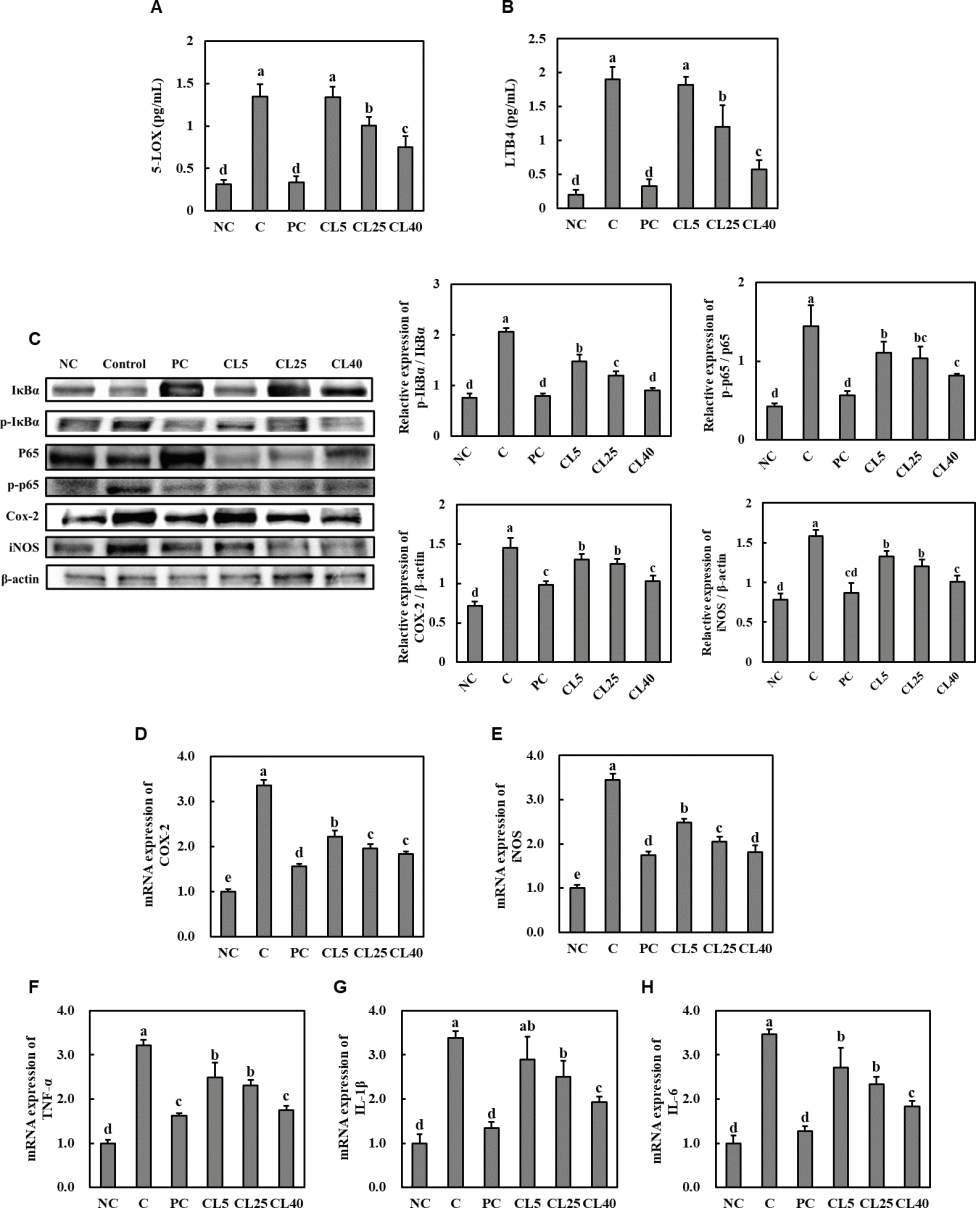
Effects of CL on the levels of 5-LOX (A) and LTB4 (B); protein expression of p-IκBα, p-p65, COX-2, and iNOS (C); and mRNA expression of *COX-2* (D), *iNOS* (E), *TNF-α* (F), *IL-1β* (G), and *IL-6* (H) in cartilage tissue from rats with MIA-induced osteoarthritis. NC: normal diet; Control (C): normal diet + MIA injection; PC: diet containing 20 mg/kg/bw ibuprofen + MIA injection; CL5: diet containing 5 mg/kg/bw CL + MIA injection; CL25: diet containing 25 mg/kg/bw CL + MIA injection; CL40: diet containing 40 mg/kg/bw CL + MIA injection. Values are presented as the mean + SD. Different letters (a > b > c > d > e) indicate significant differences with *P* < 0.05, as determined using Duncan’s multiple range test. CL = *Curcuma longa* L. extract; MIA = monosodium iodoacetate; NC = Normal Control; PC = Positive control.

MIA injection in rats led to elevated levels of 5-LOX and LTB4 (Fig. 5A and B) and protein expression of p-IκBα, p-p65, COX-2, and iNOS in cartilage tissue (Fig. 5C). Moreover, MIA injection increased the mRNA expression of *COX-2*, *iNOS*, *tumour necrosis factor-α* (*TNF-α*), *interleukin* (*IL*)-*1β*, and *IL-6* in cartilage tissue (Fig. 5D–H). However, in the PC and CL groups, MIA injection resulted in a notable reduction in the levels of 5-LOX and LTB4; protein expression of p-IκBα, p-p65, COX-2, and iNOS in cartilage tissue; and mRNA expression levels of *COX-2*, *iNOS*, *TNF-α*, *IL-1β*, and *IL-6* compared with that in the MIA control group (*P* < 0.05; Fig. 5).

### CL ameliorated the catabolic factors in cartilage tissue from rats with MIA-induced OA

**Fig. 6:**
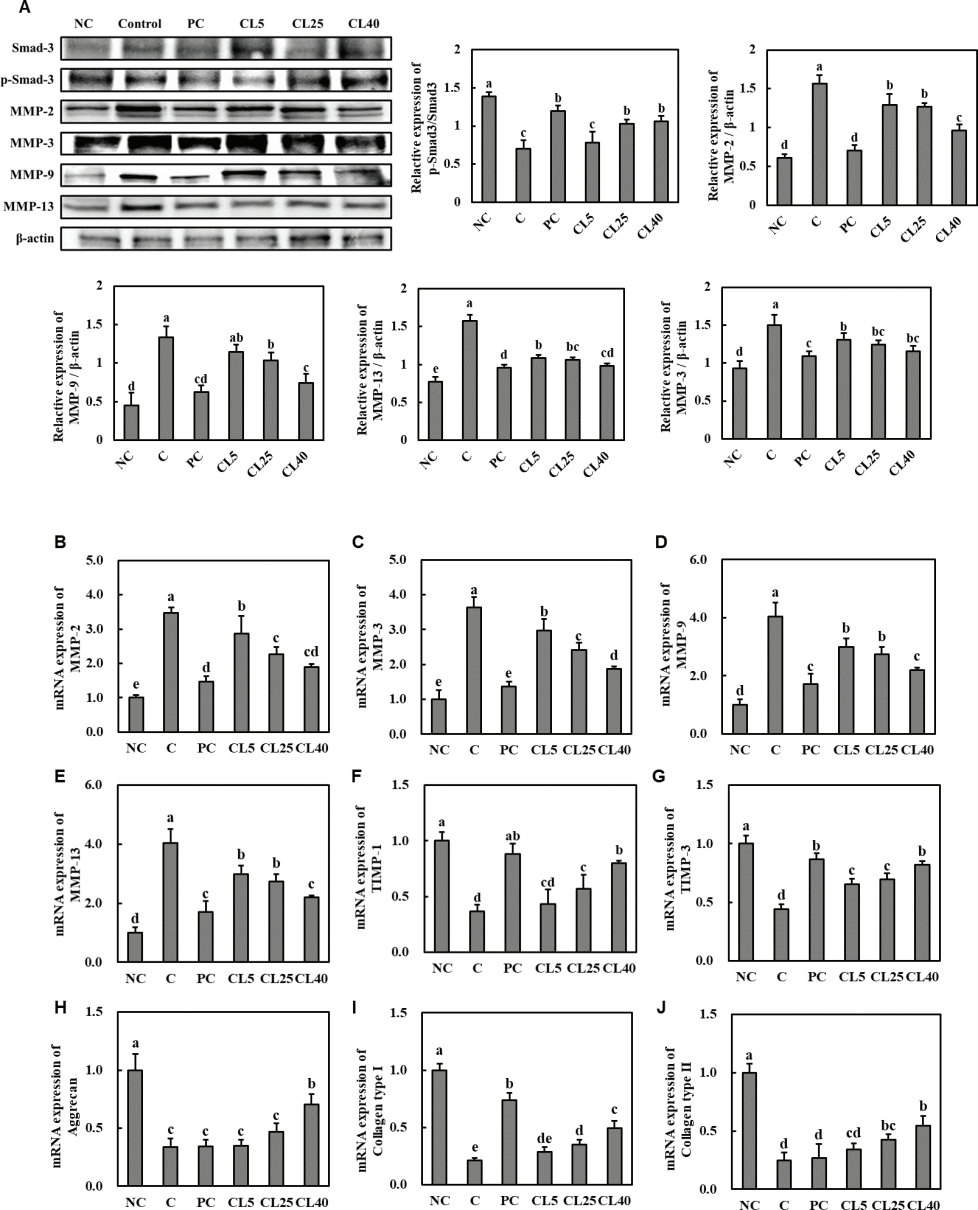
Effects of CL on the protein expression of p-Smad3 and MMPs (A) and mRNA expression of *MMP-2* (B), *MMP-3* (C), *MMP-9* (D), *MMP-13* (E), *TIMP-1* (F), *TIMP-3* (G), *aggrecan* (H), *collagen type 1* (I), and *collagen type 2* (J) in cartilage tissue from rats with MIA-induced osteoarthritis. NC: normal diet; Control (C): normal diet + MIA injection; PC: diet containing 20 mg/kg/bw ibuprofen + MIA injection; CL5: diet containing 5 mg/kg/bw CL + MIA injection; CL25: diet containing 25 mg/kg/bw CL + MIA injection; CL40: diet containing 40 mg/kg/bw CL + MIA injection. Values are presented as the mean + SD. Different letters (a > b > c > d > e) indicate significant differences with *P* < 0.05, as determined using Duncan’s multiple range test. CL = *Curcuma longa* L. extract; MMP = Matrix metalloproteinases; MIA = monosodium iodoacetate; NC = Normal Control; PC = Positive control.

We investigated the anabolic and catabolic factors in the articular cartilage of MIA-induced OA rats to elucidate the molecular mechanism underlying CL’s effect on OA. The protein expression level of p-Smad3 decreased, while that of MMP-2, MMP-3, MMP-9, and MMP-13 increased in cartilage tissue from MIA-injected rats compared with that in cartilage tissue from normal control rats. However, dietary administration of ibuprofen or CL increased the protein expression of p-Smad3 and decreased that of MMP-2, MMP-3, MMP-9, and MMP-13 in cartilage tissue compared with that of the control (*P* < 0.05; Fig. 6A).

The mRNA expression levels of *MMP-2*, *MMP-3*, *MMP-9*, and *MMP-13* significantly increased, while those of *tissue inhibitor of metalloproteinases* (*TIMP*)*-1*, *TIMP-3*, *aggrecan*, *collagen type I*, and *collagen type II* significantly decreased in cartilage tissue from MIA-injected rats compared with those in cartilage tissue from normal rats. However, the mRNA expression levels of *MMP-2*, *MMP-3*, *MMP-9*, and *MMP-13* significantly decreased, while those of *TIMP-1*, *TIMP-3*, *aggrecan*, *collagen type I*, and *collagen type II* significantly increased in the ibuprofen- and CL-treated groups compared with those in the MIA-induced OA group (*P* < 0.05; Fig. 6B–J). These findings suggest that CL potentially plays a role in the onset of degenerative OA via Smad3 activation and MMP suppression in the anabolic and catabolic pathways, respectively.

## Discussion

Interest in the use of natural products as complementary and alternative medicine for OA has grown, driven by their potentially reduced side effects and toxicity (13). In this study, we investigated the effect of CL in MIA-induced OA rats to evaluate the efficacy of novel alternative medicines in the treatment of OA. MIA, known for inhibiting glyceraldehyde-3-phosphate dehydrogenase activity (14), was used to induce OA via intra-articular injection, leading to chondrocyte death in articular cartilage. Our findings revealed that intra-articular MIA injection triggered matrix degradation and inflammation in the articular cartilage of rats. However, CL ameliorated histological and mineralisation-parameter alterations and pain intensity in rats with MIA-induced OA.

In OA, the over-activation of 5-LOX and subsequent increase in LTB4 contribute to the inflammatory cascade. The enzyme 5-LOX is responsible for the synthesis of leukotrienes, including LTB4, from arachidonic acid. LTB4 induces the recruitment of immune cells, such as neutrophils, to the inflamed joint. The accumulation of these immune cells exacerbates inflammation, leading to tissue damage and OA progression (15, 16). We observed a significant reduction in 5-LOX and LTB4 levels in the articular cartilage of CL-treated rats compared with that in control rats. Additionally, CL administration decreased serum PGE_2_and NO levels as well as the protein expression of p-IκBα, p-p65, COX-2, and iNOS in the cartilage of MIA-injected rats. Moreover, it also reduced the mRNA expression levels of *COX-2*, *iNOS*, *TNF-α*, *IL-1β*, and *IL-6* in the cartilage of MIA-injected rats. These findings further corroborate the notion that CL exhibits anti-inflammatory properties for the treatment of OA.

OA is characterised by a progressive deterioration of the ECM of articular cartilage, primarily orchestrated by MMPs. The ECM predominantly comprises collagen, constituting approximately 30% of the body’s total protein, and aggrecan, a crucial proteoglycan essential for the normal function of joints. The structural integrity and proper functioning of articular cartilage rely on key factors, such as aggrecan and collagen, within the ECM. The Smad signalling pathway and TIMPs are pivotal MMP inhibitors in chondrocytes (17–22). We observed increases in the protein and mRNA expression levels of MMPs in the articular cartilage of OA rats. Conversely, the expression of p-Smad3, TIMP-1, TIMP-3, aggrecan, collagen type I, and collagen type II decreased in the articular cartilage of OA rats. In CL-treated OA rats, MMPs decreased, while p-Smad3, TIMPs, aggrecan, collagen type I, and collagen type II increased. These results suggest that CL effectively suppresses the degradation of articular cartilage in OA.

Curcumin derived from CL has been established to exhibit multifaceted actions in the pathogenesis of OA. Notably, studies by Mathy-Hartertet et al. (23) have suggested the effectiveness of curcumin in treating OA by inhibiting the production of inflammatory and catabolic mediators in chondrocytes. Additionally, Li et al. (24) demonstrated that curcumin exerts inhibitory effects on apoptosis and inflammatory signalling by modulating extracellular signal-regulated kinase 1/2-induced autophagy in chondrocytes. In our study, the efficacy of CL was evaluated in animal models, and CL was found to exhibit significant pain suppression and joint-damage inhibition in OA rats. Previous findings and our results collectively indicate that curcumin possesses the potential to directly impact chondrocytes, suggesting a plausible role in OA treatment via its anti-inflammatory and cytoprotective functions. However, numerous studies and more extensive clinical trials are still required to definitively recommend curcumin as an alternative treatment. In conclusion, our findings suggest that CL supplementation may prevent OA development and exhibit effectiveness in OA therapies.

## Conflict of interest and funding

The authors have no conflicts of interest to declare. No funding was received.
